# Health workforce capacity of intensive care units in the Eastern Mediterranean Region

**DOI:** 10.1371/journal.pone.0286980

**Published:** 2023-06-16

**Authors:** Arooj Jalal, Kazuyo Iwamoto, Gulin Gedik, Hamid Ravaghi, Chiori Kodama

**Affiliations:** 1 Health Workforce Development, World Health Organization Regional Office for Eastern Mediterranean, Cairo, Egypt; 2 Health Emergencies Programme, World Health Organization Regional Office for Eastern Mediterranean, Cairo, Egypt; 3 Hospital Care Management, World Health Organization Regional Office for Eastern Mediterranean, Cairo, Egypt; University of Sharjah College of Health Sciences, UNITED ARAB EMIRATES

## Abstract

**Objective:**

The onset of COVID-19 pandemic increased the need for functioning and equipped intensive care units (ICUs) with staff trained in operating them. In the Eastern Mediterranean Region, this also triggered the need for assessing the available capacities of ICUs and health workforce so that appropriate strategies can be developed to address emerging challenges of staff shortages in the wake of COVID-19. To address this need, a scoping review on the health workforce capacity of intensive care units in the Eastern Mediterranean Region was conducted.

**Methods:**

A scoping review methodology as outlined by Cochrane was followed. Available literature and different data sources were reviewed. Database includes Pubmed (medline,Plos included), IMEMR, Google Scholar for peer-reviewed literature, and Google for grey literature such as relevant website of ministries, national and international organization. The search was performed for publications on intensive care unit health workers for each of the EMR countries in the past 10 years (2011–2021). Data from included studies was charted, analysed and reported in a narrative format. A brief country survey was also conducted to supplement the findings of the review. It included quantitative and qualitative questions about number of ICU beds, physicians and nurses, training programs as well as challenges faced by ICU health workforce.

**Results:**

Despite limited data availability, this scoping review was able to capture information important for the Eastern Mediterranean Region. Following major themes appeared in findings and results were synthesized for each category: facility and staffing, training and qualification, working conditions/environment and performance appraisal. Shortage of intensive care specialist physicians and nurses were in majority of countries. Some countries offer training programmes, mostly for physicians, at post-graduate level and through short courses. High level of workload, emotional and physical burnout and stress were a consistent finding across all countries. Gaps in knowledge were found regarding procedures common for managing critically ill patients as well as lack of compliance with guidelines and recommendations.

**Conclusion:**

The literature on ICU capacities in EMR is limited, however, our study identified valuable information on health workforce capacity of ICUs in the region. While well-structured, up-to-date, comprehensive and national representative data is still lacking in literature and in countries, there is a clearly emerging need for scaling up the health workforce capacities of ICUs in EMR. Further research is necessary to understand the situation of ICU capacity in EMR. Plans and efforts should be made to build current and future health workforce.

## Introduction

Many developing countries had been facing a continued shortage of health workers and intensive care unit (ICU) facilities when the onset of COVID-19 pandemic significantly impacted delivery of health services across the world and increased the need for functioning and equipped ICUs with staff trained in operating them. This also triggered the need for assessing the available capacities of ICUs and health workforce so that appropriate strategies can be developed to address emerging challenges of facility and staff shortages in the wake of COVID-19 [1].

ICU provide patient care and treatment needed for critically ill patients. A consolidated, universal agreement however, of what constitutes an ICU is not available. Fundamentally, an ICU needs to be a well-organized combination of a well-equipped facility, infrastructure, equipment and a competent multidisciplinary team working within a structure based on technical standards and best practices [1]. The definition of an ICU and therefore their beds and capacities vary from country to country. This is a significant challenge when assessing resources and capacities in a country as well as comparing them with others [1].

The Eastern Mediterranean Region (EMR) has 22 member states, majority of which are low- and middle-income countries, and there is a very steep difference between the intensive care capacity of resource rich and resource poor countries within the region. The challenge is sparsity of national level data on ICU beds and qualified specialist physicians and nurses in acquiring a comprehensive and accurate assessment of local capacities. Table 1 indicates to the ICU capacity of countries from different income levels in comparison to some EMR countries where such information is available [2, 3].

**Table 1:** ICU bed per 100,000 population in high, low- and middle-income countries.

Country	ICU bed per 100,000 population
High income	
Germany	33.9
Saudi Arabia	22.8
Oman	14.6
Canada	12.9
England	10.5
Upper middle income	
South Africa	8.9
Iran	4.6
Malaysia	3.4
Low and lower-middle income	
Indonesia	2.7
Sri Lanka	2.3
Pakistan	1.5
Uganda	0.1

Similarly, the optimal model for staffing ICUs is still unclear and any recommendations available varies for different ICUs models such as high and low intensity ICUs. Depending on the ICU capacity, the optimum physician-to-patient ratio may vary but the World Federation of Societies of Intensive and Critical Care Medicine (WFSICCM) recommends that a ratio of at least 1:8 specialist physician or intensivist is preferable and should not be lower that 1:15, findings consistent with other literature [4, 5]. There is also an ongoing debate about whether or not round the clock presence of specialist physicians is necessary in these units, and while there is a considerable staffing cost and physician shortages to consider, the benefits do seems to outweigh the costs in high-volume, high-acuity ICUs [6].

Equally critical is the presence of specially trained nurses in the ICU. National recommendations for developed countries such UK and Australia require a minimum nurse to patient ratio of 1:1 for ventilated and other critically ill patients and 1:2 nursing staff for patients determine to be less critical [5, 7]. As the ideal is often not possible in low resource settings, nurses are frequently left in charge of the patients in ICUs with nurse-to-patient ratio of 1:4 or even lower [8]. In any case, the number of staff required should be calculated according to the number of ICU beds, number of shifts per day, occupancy rate and overall level of ICU such as level 1 where patients do not require organ support or level 3 where patients require mechanical ventilation [9].

In the current context of the pandemic, the importance of timely access to emergency, critical and intensive care is being emphasized by countries and regions and the need for providing these services through integrated approaches. It is of interest to note that the World Health Organization (WHO) is responding to these calls by planning to support countries in strengthening their health service delivery through ECO- systems (emergency, operative and critical care services). It is a mechanism that ensures these services are accessible to people who need them through integrated planning that places longitudinal primary care at the centre of this system [10]. Research about intensive care is also low in the Eastern Mediterranean Region as in many developing countries [11, 12]. A systematic review on intensive care unit capacity in 36 low-income countries found 50% of them did not have any published data on ICU capacity [13]. Therefore, in order to improve knowledge about the health workforce capacity of intensive care units in EMR, a scoping review of available literature and different data sources in the region was conducted. A brief country survey was also conducted to supplement the findings of the review. The survey aimed to capture the current capacities of ICU beds and health workforce involved in dealing with COVID-19 patients.

While the terms ICU and critical care may be used interchangeably in literature, critical care can often extend beyond intensive care units. This paper focuses on health workforce capacity of intensive care units and the term ICU will be used for consistency.

### Objectives

- Provide an up-to-date and comprehensive overview of the current capacity of ICU workforce in the EMR member states.
- Reviewing the impact of COVID-19 on ICU capacity and the workforce in EMR.

## Methods

### Identifying relevant studies

A scoping review methodology as outlined by Cochrane and Joanna Briggs Institute manual was followed [14, 15]. Two individuals independently searched four databases. Searches for peer reviewed literature included Pubmed (medline,Plos included) IMEMR Google Scholar and Google search engine). Grey literature was sought from Google as well as relevant website of ministries, national and international organization. The search was performed for publications on intensive care unit health workers for each of the EMR countries in the past 10 years, by combining search terms as well as free text depending on the database used, with no language restriction. Search terms used included “intensive care unit” “critical care” “intensive care” “icu” “workforce” “health workforce” “health care workers” “staff” “healthcare professionals” “physicians” “nurses” “developing countries” “Eastern Mediterranean”. A 10-year search timeline between 2011 and 2021 was employed to include the most recent information and evidence in the region.

**Fig 1:**
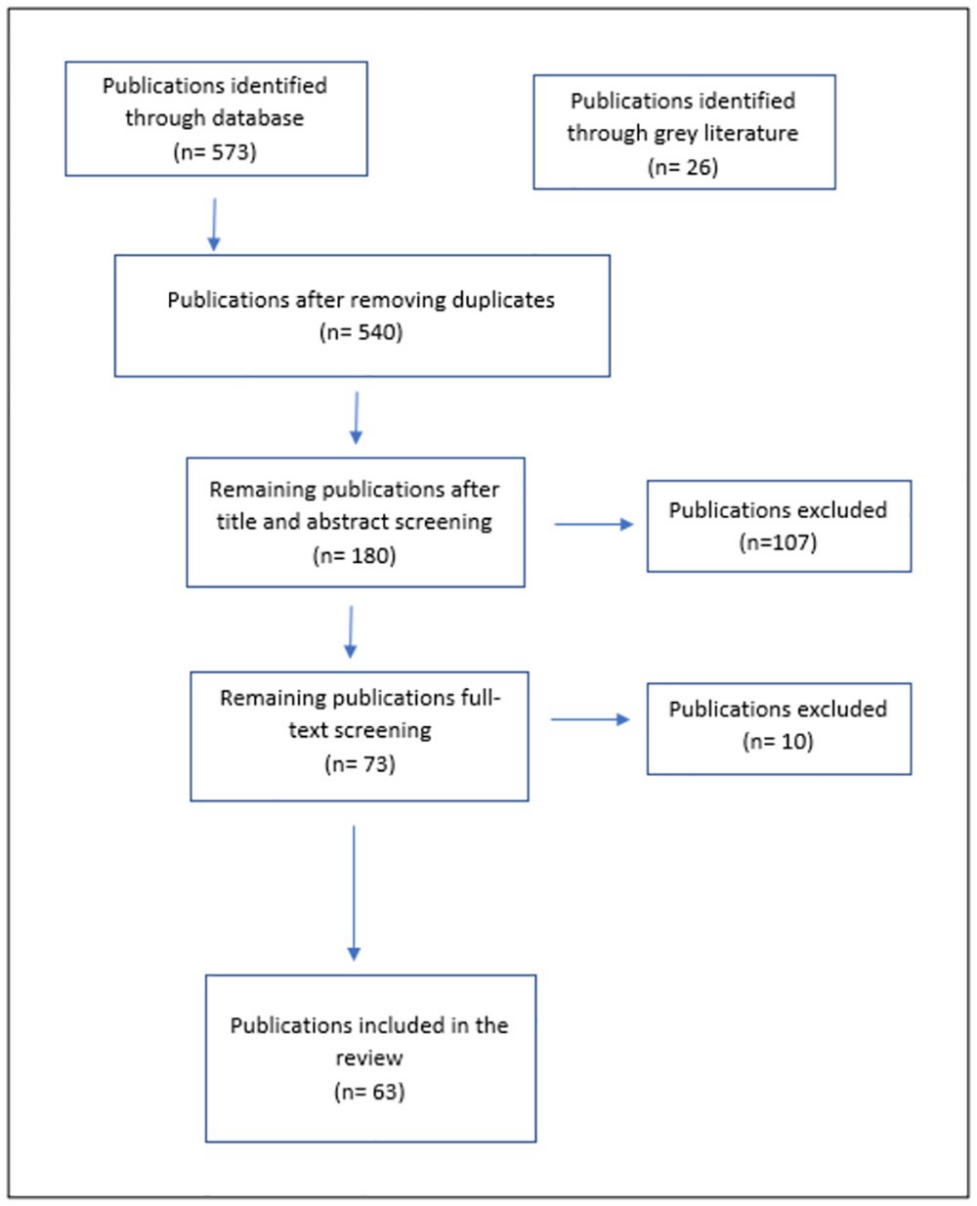
Flow diagram of included articles.

Eligible articles were those that included critical care or ICU setting, bed numbers, staffing capacity, ICU training and qualification and performance appraisal. Articles that did not include health workers working in critical or intensive care unit settings were excluded, as well as those not published within the selected time period and those for which full text was not available. Additionally, reference lists of included articles were also reviewed to identify relevant literature. Mendeley was used to facilitate the screening process. Initial search yielded 599 titles, after removing duplicates and screening titles, 73 articles were screened for full text after which 63 were available for inclusion based on the inclusion criteria (Fig 1). In addition to that, after the data extraction from selected articles was completed using the data extraction chart, another screening was performed of all entries to exclude any studies not fitting the inclusion criteria.

Limitations included consulting limited number of databases and reviewing a limited number of journals. Certain articles may not have been retrieved if the name of the country was not mentioned in the title or abstract. This could have resulted in an underestimation of the number of publications on ICU health workforce in the Eastern Mediterranean Region.

### Charting data

An excel spreadsheet was used to extract the data from 63 articles. The data extraction chart was developed with the consideration of the type of information to be extracted from the included literature to answer the research questions. It included title of the article, name of authors, journal and publishing date, type, aims and objectives of the study, countries the study is based in, design/methodology, findings, aspects of HWF related to ICU capacity and limitations of the study if reported.

### Collating, summarising and reporting results

Data such as date of publication, authors, country of the study etc are straightforward to extract and allow for a description of papers included in the scoping review. Data from the included literature was reviewed and information most relevant to answer the review questions was extracted and charted it in the excel sheet. Scoping review guidelines do not call for analysis beyond basic descriptive analysis of content such as frequency counts of concepts, populations etc. [16, 17] Information is synthesized descriptively and can be presented in categories or themes. A formal thematic analysis is beyond the scope of scoping reviews [18]. Upon review of the included articles, the extracted information was grouped under major themes, which were facility and staffing, training and qualification, working conditions/environment and performance appraisal. The results were grouped under these themes and re-analysed within each theme and if required content was moved within themes for a bitter fit. As the number of studies was limited, we included all the studies which were then carefully interpreted and reported. Quality assessment was not performed due to limited number of studies; therefore, all studies were included, and all data is reported in a narrative format.

### Country survey

The questionnaire included quantitative and qualitative questions about number of ICU beds, ICU physicians and nurses, national training programs as well as challenges faced for ICU health workforce. Responses were received from six countries with national level reporting from Jordan, Afghanistan, and Pakistan. Palestine reported data for West Bank only and facility level data representative of ICUs across the country was provided by Djibouti and Oman and will be considered as representative at national level in this analysis.

An ethics statement is not applicable as this study is based on published literature and data which is publicly available within countries and/or government agencies.

## Results

### Study characteristics

Table 2 contains the number and type of publications included from each country. Majority of the articles were from Egypt, Iran, Jordan, and Saudi Arabia and focused on appraisal of certain practices and knowledge. Most publications (60% were cross-sectional surveys, only 4.7% were a qualitative examination of the subject and 20% were reviews or reports. Afghanistan, Bahrain, Djibouti, Egypt, Iran, Iraq, Kuwait, Lebanon, Sudan (40%) had facility level studies. Djibouti, Libya, and Morocco (13%) were also part of regional level studies while Jordan, Somalia as well as Libya (13%) had both national and facility level studies. National level studies were retrieved for Saudi Arabia, Pakistan, Oman, Syria, Tunisia, and Yemen (27%). No publications were found for UAE.

**Table 2:** Number and type of publications from EMR countries included in the scoping review.

Country	Level of studies	Total Number of studies included	Theme			
			Staffing	Training	Working environment/working conditions	Performance appraisal/competency
Afghanistan	Facility level	1	1			
Bahrain	Facility level	2	1		1	
Djibouti	Regional level	1	1			
Egypt	facility level	16	3	4	4	5
Iran	Facility level	13		3	6	4
Iraq	facility level	1				1
Jordan	National+facility level	9	1		2	6
Kuwait	Facility level	1				1
Lebanon	Facility level	1			1	
Libya	facility level+regional level	3	2		1	
Morocco	Regional study	1	1			
KSA	National level	5	3	1	1	
Pakistan	National level	2	1	1		
Oman	National level	1	1		
Somalia	National+facility level	2	1		1	
Sudan	Facility level	1	1			
Syria	National level	1		1		
Tunisia	National level	1		1		
UAE	None	0				
Yemen	National level	1	1			
		63	18	11	17	17

### Facility and staffing

**Fig 2:**
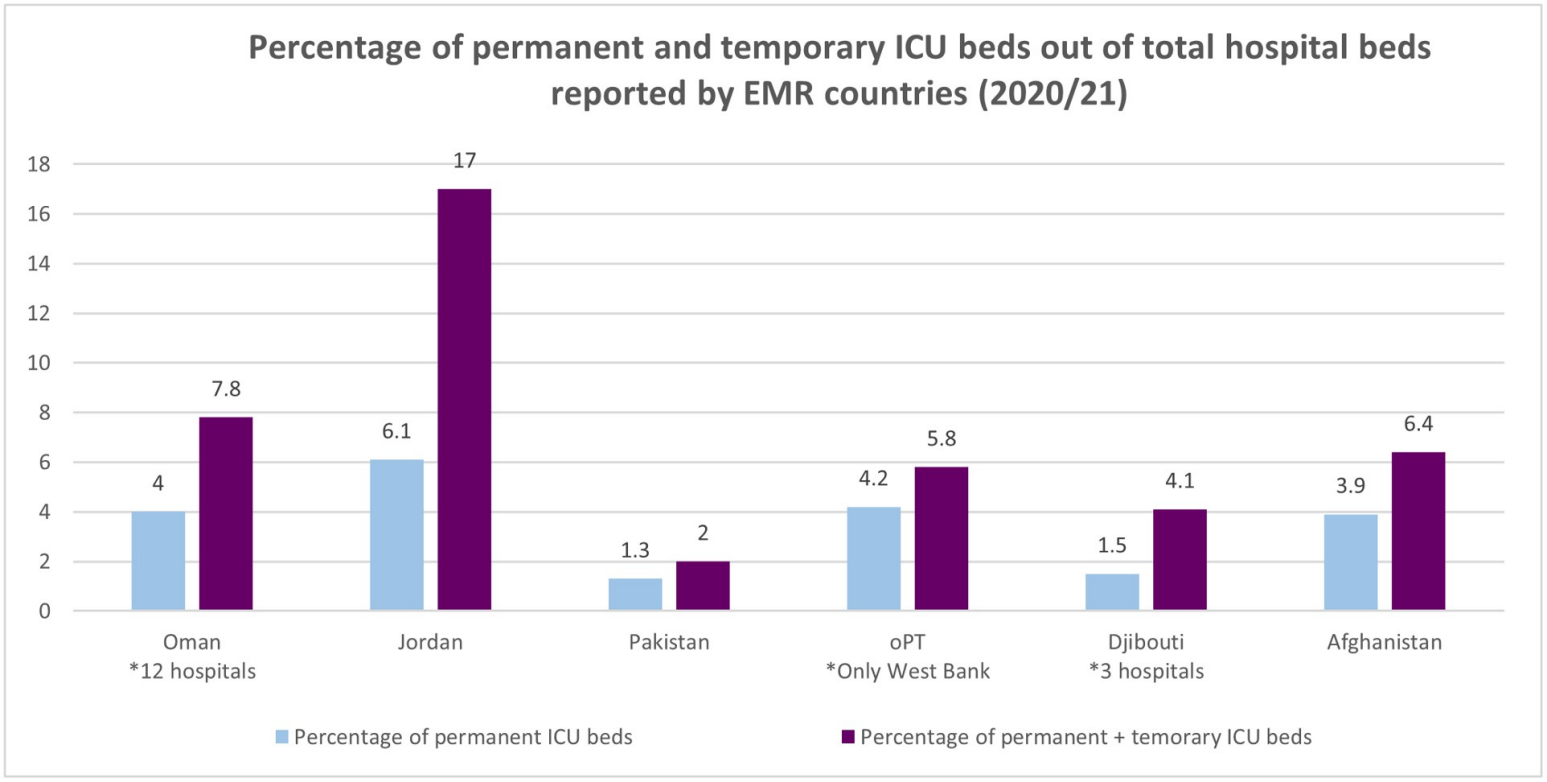
Percentage of permanent and temporary ICU beds out of total hospital beds reported by EMR countries (2020/21).

Comprehensive and accurate data on facility and staffing can be vital for planning and providing quality healthcare. In this scoping review, very few studies had estimates of ICU beds in a country. However, with the onset of the pandemic, increase in ICU beds across countries was reported to cope with the overwhelming number of cases requiring intensive care due to COVID-19 infections. Survey findings also demonstrated the difference in actual and surge capacity as hospitals beds and other resources in hospitals were converted into flexible ICU beds for treatment of COVID-19 patients. Some countries responding to the survey reported nearly doubling their ICU capacity (Fig 2): Percentage of permanent ICU beds out of total hospital beds in Jordan was 6.1 which increased to 17 percent when accounting for the additional ICU beds converted for COVID-19 response [19] Similarly in Oman, permanent ICU beds made up 4 percent of total hospital beds while additional ICU beds raised the proportion to 7.8 percent. Afghanistan reported an increase in its ICU beds from 3.9 percent to 6.4 percent. In other countries, the increase with additional ICU beds was a little lower. Example of Pakistan, reporting an increase from ICU beds comprising 1.3 percent of total beds to 2 percent. West Bank increased its ICU beds from 4.2 to 5.8 percent.

Health worker shortages were referred to in literature but exact numbers of ICU health workforce at the national level were not available for many EMR countries and physician and nurse to patient ratio were mostly reported for facility level [20] Most of the data found in literature was related to anaesthesia physicians as it is common in many countries for anaesthesiologist/anaesthesia physicians to run intensive care units or serve as consultant physicians.

In the Tunisian health system, public sector medical ICUs were generally managed by medical intensivists and surgical field managed by anaesthesiologists. Private sector ICUs usually managed by anaesthesiologists recruited on a full-time basis [21] One of the studies on Africa’s critical care capacity before the COVID-19 pandemic, published in 2020, reported density of anaesthesia physicians in Djibouti and Egypt to be 1.01 per 100,000 population and 6.01 per 100,000 population respectively [22]

A survey performed in 2020, of 16 hospitals in Libya, found that intensive care specialists were available 24 hours a day in nine (56.3%) hospitals, four hospitals had them on call for 24 hours a day and three hospitals didn’t have the 24 hours presence of such specialists. Nurse-to-patient ratio ranged from 1:1 in some ICUs to 1:4 in other [23] The Lebanese Critical Care Society approved by the Lebanese Order of Physicians has over 100 critical care physicians in its membership from different specialties such as pulmonary, anaesthesiology, internal medicine—which may be taken as in indication to their number [24].

The country survey also showed that majority of intensive care related specialists were qualified as anaesthesiologists followed by internal medicine. In all six responding countries qualified intensivists comprised of very low numbers ranging from none in Afghanistan to 1 in West Bank, 2 in Jordan, 8 in Pakistan, and 49 in Oman (data from 2020–2021). Afghanistan also had one of the lowest availabilities of ICU staff reported by the countries with only one physician per 5–10 beds and one nurse per 3–5 beds. Djibouti reported 1 physician for 6 beds and one nurse for 3 beds, Jordan reported nurse to bed ration ranging from 1 per 4 beds to 1 per 5–8 beds while West Bank and Pakistan reported non-availability of such numbers.

Nurse-to-patient ratio in a Moroccan tertiary care ICU was found to be 1:2 during the day and night shifts [25]. A national level study conducted in 151 hospital ICUs in Pakistan found 1:1 nurse to bed ratio during the day was only available in 53.5 percent of units, dropping to 47.8 percent during the night shift. A senior clinician trained in intensive care was available in only 12.1 percent of ICUs. For Jordan nurse-to-patient ratio reported in the country survey ranged from 1:4 in JUH, 1:5 in RMS and KAUH to 1:8 in MoH. Overall, severe staff shortages were reported in the survey, especially of physicians and nurses trained in intensive care.

### Training and qualification

Training of health workforce for intensive care differs across countries and is done through a variety of programmes including master’s degree, post-graduate training, diplomas, and short courses. Literature on training of doctors, nurses and physiotherapists were found in Bahrain, Egypt Iran, Iraq, Jordan, Libya, Morocco, KSA, Pakistan, Syria, and Tunisia. Further information of available training programs in Afghanistan, Pakistan, Oman, Jordan, and Palestine was also obtained through the country survey.

Several countries have post-graduate clinical training programmes which are generally 4 to 5 years in length as shown by the findings from the scoping review and country survey. These include a 5 year-critical care residency programme for physicians offered by The Saudi Board of Critical Medicine and a 4-year critical care medicine fellowship for physicians in Pakistan under College of Physicians and Surgeons (CPSP) [26, 27]. Graduates in Tunisia have two pathways for specialization in critical care: a 5-year curriculum of anaesthesiology and surgical intensive care medicine or 4 years in medical intensive care medicine [21]. Graduates in Libya can specialize in anaesthesia and intensive care under the Libyan Medical Specialties Board as well as from Arab Board of Health Specializations in Anaesthesia and Intensive care [28]. The Arab Board of Medical Specialization was founded by the Council of Arab Health Minister of the Arab League and is responsible for establishing certification procedures for a wide range of medical specialties in coordination with teaching institutions in its member countries [29].

Jordan not only has a residency program by the Department of Anaesthesia and Intensive Care at Jordan University Hospital (JUH) but the Ministry of Health also sends it ICU, Respiratory and Pulmonary physicians to JUH for a 2–3 year specialization as part of its CPD program [19, 30].

Specialization through master’s degree is also an option in some countries though few appeared in the review search such as master’s degrees in intensive or critical care available for nurses in Bahrain, Iran, and Jordan [31, 32]. Literature for Jordan also indicated presence of MSc and doctorate for ICU physical therapists [33]. Several short courses are also offered for physicians and nurses in the region such as a 9-month diploma in critical care for bachelor’s degree holder in Jordan and a Post-RN diploma in emergency and critical nurses in Pakistan. Oman also offers critical care diplomas for nurses in adult critical care and fundamental critical care support [19]. In Libya, the Centre of Development of Medical Manpower plays a major role in continuing medical education and conducts 18-month training sessions for specialized qualification in intensive care [28].

### Working conditions/environment

Working conditions and environment can have a notable impact on performance, motivation, turnover, and job satisfaction. 16 studies on related topics were found from Bahrain, Egypt, Iran, Jordan, and Libya. High level of workload and stress is a consistent finding across all countries often resulting from shortage of available staff. 57.1% of intensive care nurses working at a Bahraini hospital were uncertain if they wanted to continue working in ICU and only 21.4% were satisfied working there [34]. 68.2% of ICU health workers in an Egyptian study reported moderate burnout and over 50% of nurses had high level of emotional exhaustion compared to 38.8% of physicians [35]. Similarly, 96% of ICU nurses in a study from Iran had high risk level for secondary traumatic stress and 42% for burnout [36]. Jordanian ICU nurses were found dealing with higher job stressor compared to nurses in other wards and a cross-sectional study in two Somalian hospitals found work overload, role ambiguity and conflict were significant stressors for ICU staff [37, 38]. Survey findings corroborated with these results with all countries reporting very high workload, high turnover, severe shortage of physicians and nurses specialized in intensive care, especially exacerbated due to COVID-19. Jordan’s survey response highlighted that in the beginning on the pandemic some staff members had to stay in health facilities for over a month at a time in addition to the risk of getting infected. Several countries also mentioned the continuous rotation of limited number of specialists being rotated between ICU and other departments results in disruption of availability in some departments.

### Performance appraisal

Performance appraisal is especially important in healthcare settings to identify learning and continuing development needs and demonstrate competency in practice among other benefits. A total of 17 studies evaluated competency and performance of ICU health workers and nearly all of them were focused on nurses. Gaps in knowledge were found regarding importance of reporting medication errors, management of critically ill patients, stroke and ventilator associated pneumonia in studies from Egypt, Iran, Jordan, and Kuwait. In an Iranian PICU, 74.8% of neonates experienced at least one medication error, 57% made by physicians and 43% by nurses [39]. 56.4% of critical care nurses in a Kuwaiti hospital passed the skills exam for CPR while only 15.7% passed the knowledge test. Jordanian studies indicated that failure to report medical errors was largely because nurses did not think the errors were serious enough [40]. Studies based in Egypt, Jordan, Saudi Arabia and Iran found ICU nurses to be not fully compliant to critical care guidelines and recommendation for procedures like endotracheal intubation and one of the main reasons were lack of time [41–48]. It was also demonstrated that performance of ICU nurses improved when exposed to focused training courses [49, 50].

## Discussion

While, in many countries, respective national societies of intensive care collect data to capture the ICU capacity in their countries which can be publicly accessible, this was not the case in EMR [51]. Moreover, studies focusing on staffing were mostly limited to facility level which is not necessarily nationally representative especially in countries with a sharp rural-urban or geographical disparity in availability of resources. Difference between the availability of the ICU resources between urban and rural areas, which is important in terms of equal access the ICU care, could not be captured in this review. Regarding to the nurse-to-patient ratio at ICU in EMR (1:1 to 1:8), it has less capacity compare to Asian countries (1:1 to 1:3) [52] Scaling up of resources in response to the COVID-19 pandemic was observed however, it raised an important point of concern about how countries managed to increase workforce capacity in intensive care for treating COVID-19 patients in a short period of time and how that may impact quality of care. At global level, it has findings that anaesthesiologists play a central role in ICU in providing patient safety and performance improvement due to their strength in sedation procedure, mechanical usage, and rapid response, and this practice is commonly seen in EMR as well [53]. Similar management of ICUs was found in African regions, such as South Africa [54]. Increase in ICU beds and admissions also meant severe shortage of physicians and nurses, especially ICU specialists, continuous rotation of limited number of specialists in ICU departments and significant increase in workload. Relying on fragmented data for planning to overcome health workforce shortages and other needs of health systems can result in weak policies and delay progress. Obtaining accurate and comprehensive baseline data and establishing up-to-date data bases of critical care workforce is therefor, essential for evidence-based policy decisions.

None of the included studies explored the presence of intensive care training programmes but rather other aspects and impact of training programmes. Ideally, the healthcare workers would be qualified and certified in the field of intensive care. However, a limited number of countries (Bahrain, Egypt, Iran, Jordan, Pakistan, Syria, Tunisia, Saudi Arabia) have established a training program at the post-graduate level to train ICU physicians, nurses, and other health workers. Previous research has suggested that having a certified nurse specialist in intensive care with advanced skills as a head nurse in the ICU may help to improve patient outcomes by leading the team to optimal solutions [55]. There are several established training programs such as a five-year residency program for physicians in Saudi Arabia and a four-year fellowship in Pakistan in the literature, a master’s degree in intensive care for nurses in Jordan and the critical care diploma for nursing in Oman in the survey. In contrast, survey, and literature findings both found accounts of lack or absence of training programmes, curriculum’s insufficiency and ineffectiveness, inefficient planning, ineffective implementation, and lack of correlation between job description and curriculum. Such shortcomings in education and training programmes are a challenge in producing a well-trained and high-quality workforce. This in-turn has implications on the delivery of required health services, quality of care and overall performance of health system. Countries in the region must invest in establishing and scaling up relevant post-graduate and specialty programmes with uniform curricula and ensure their implementation. Equally necessary is access and availability of continuous professional development opportunities for ICU workforce.

Majority of studies grouped under the theme of working conditions and environment have looked into mental health issues and job satisfaction among personnel working in ICUs. As mentioned earlier and consistent with findings from other studies, health workers stationed in ICUs work under significantly higher stress and trauma risk factors compared to those in other hospital wards or units. Nearly all the studies in the review focused on nurses working in ICUs and it is clear they work under not just physical burnout but also emotional exhaustion. The issue of burnout among the ICU staffs is widely observed in multiple countries outside of the EMR as well. For example, a systematic review of burnout in ICU professionals revealed the prevalence ranged from 6–7% in 25 countries [56]. Gaps in knowledge about many basic procedures performed in ICUs, lack of awareness about the importance of reporting medical errors and poor adherence to recommended guidelines reflects the need of continued medical education, increased active learning instead of lecture-based and better engagement with the health workers for an improved understanding of their needs and experiences. Studies also showed that active learning and continued education does improve the performance of ICU staff and adherence to guidelines which is an important insight for preparing a competent health workforce equipped to deal with ongoing and future public health emergencies. All the findings discussed above imply safety and quality concerns in health services. Increased workload as a result of staff shortages limit the ability of critical care nurses to deliver quality patient care and increases the risk of infections and decreases positive outcomes for critically ill patients [57, 58] Working under physical and emotional burnout, and inadequate knowledge of basic critical care procedures increases risk of medical errors, poor adherence to recommended guidelines, puts the safety of patients and health workers at risk and has negatively affects the quality of care [59].

## Conclusion

Although the literature on ICU and ICU capacities in the Eastern Mediterranean Region was found to be limited, it did provide valuable information on certain aspects of ICU health workforce capacity. Despite the absence of baseline data on ICU capacities before the COVID-19 pandemic, it is clear that many countries in the region struggle with challenges of increased workload, mental health stressors, training, and competency of their health workers in ICUs, exacerbated further with the onset of COVID-19 pandemic. Well-structured, up-to-date, comprehensive and national representative data is still lacking in literature and in countries. This is particularly relevant to ICU bed capacity, number of qualified and trained staff available and ratio of physicians and nurses to ICU beds. It is apparent from the fragmented information in literature that there must be a functioning health workforce information system with adequate disaggregation in all countries which will provide crucial information for capacity planning and resource allocation.

The need for scaling up health workforce capacities of ICUs is a clearly emerging need in EMR. The plans and efforts should be made to build current and future health workforce. In the short-term, countries may repurpose some of the existing health workforce with shorter training programmes while establishing and scaling up relevant post-graduate and specialty programmes for health professionals, especially for physicians and nurses. Simultaneously, continuous professional development opportunities should be made available to current and future ICU workforce.

With the challenges mentioned it is also equally important that interventions to increase the resilience of health workers to phycological burden and workload stresses are in place as well as reducing the workload with appropriate health workforce planning. As countries in the Eastern Mediterranean region continue to battle COVID-19 while many face existing challenges with limited resources, complex emergencies and protracted crisis, initiating a systematic and sustainable health system strengthening in critical care and ICU is urgently needed with full engagement from the regional and global community.

## Supporting information

S1 Checklist PLOSONE ICU HWF PRISMA-ScR-Fillable-Checklist_2022-05-26. (DOCX)

S1 Data PLOSONE ICU HWF data extraction chart 2022-05-31. (XLSX)

S1 File PLOSONE ICU HWF Electronic search 2022-05-31. (DOCX)

## Abbreviations

CPD: Continued Professional Development
EMR: The Eastern Mediterranean Region
ICU: Intensive care unit
IMEMR: Index Medicus for the Eastern Mediterranean Region
JUH: Jordan University Hospital
KAUH: King Abdullah University Hospital
RMS: Royal Medical Services
WFSICCM: World Federation of Societies of Intensive and Critical Care Medicine

## References

[1] Kodama C, Kuniyohsi G, Abubakar A. Lessons learned during COVID-19: Building critical care/ICU capacity for resource limited countries with complex emergencies in the World Health Organization Eastern Mediterranean Region. J Glob Health 2021,11:03088. J Glob Health. 2021;: p. 11:03088. doi: 10.7189/jogh.11.03088 34326987 PMC8286446

[2] Phua J, Faruq MO, Kulkarni AP, Redjeki IS, Detleuxay K, Mendsaikhan N. Critical Care Bed Capacity in Asian Countries and Regions. Crit Care Med. 2020;(654–62). doi: 10.1097/CCM.0000000000004222 31923030

[3] OECD. Intensive care beds capacity. [Online].; 2020 [cited 2022 25. https://www.oecd.org/coronavirus/en/data-insights/intensive-care-beds-capacity.

[4] Amin P, Fox-Robichaud A, Divatia JV, Pelosi P, Altintas D, Eryüksel E, et al. The intensive care unit specialist: report from the Task Force of World Federation of Societies of Intensive and Critical Care Medicin. Joural of Critical Care. 2016 Oct; 1(35). 10.1016/j.jcrc.2016.06.001 27444985

[5] Minimum standards for intensice care units. [Online].; 2011 [cited 2022 04 25. https://www.cicm.org.au/CICM_Media/CICMSite/CICM-Website/Resources/ProfessionalDocuments/IC-1-Minimum-Standards-for-Intensive-Care-Units.pdf.

[6] Masud F, Lam TY, Fatima S. Is 24/7 in-house intensivist staffing necessary in the intensive care unit? 2018 Apr;14(2):134. Methodist DeBakey cardiovascular journal. 2018 April; 14(2)(134). doi: 10.14797/mdcj-14-2-134 29977470 PMC6027728

[7] Royal College of Nursing. [Online].; 2020 [cited 2022 March 3. https://www.rcn.org.uk/news-and-events/news/uk-nurse-staffing-ratios-in-icu-revised-to-help-manage-second-surge-of-covid-19-131120.

[8] B T. Critical care in low-income countries. Trop Med Int Heal. 2009 Feb; 14(2). 10.1111/j.1365-3156.2008.02202.x 19207174

[9] Valentin A, Ferdinande P. Recommendations on basic requirements for intensive care units: structural and organizational aspects. Intensive care medicine. 2011 October; 3(7). doi: 10.1007/s00134-011-2300-7 21918847

[10] Reynolds TA, Guisset AL, Dalil S, Relan P, Barkley S, & Kelley E. Emergency, critical and operative care services for effective primary care. Bulletin of the World Health Organization. 2020;: p. 98(11), 728–728A. doi: 10.2471/BLT.20.280016 33177766 PMC7607458

[11] Nazer LH, Kleinpell R, Olsen K, Hawari F. Capacity building for research in critical care: A pilot program in the eastern mediterranean region. Critical Care Explorations. 2021 Jan; 3(1). doi: 10.1097/CCE.0000000000000315 33458683 PMC7803936

[12] Elaibaid M, Nazer LH, Shaikha L, Al-Qadheeb N, Kleinpell R, Olsen KM, et al. Evaluating the published critical care research from the World Health Organization Eastern Mediterranean region. BMC Research Notes. 2019 December; 12(1). doi: 10.1186/s13104-019-4093-7 30658704 PMC6339311

[13] Melchiorre MG, Chiatti C, Lamura G, Torres-Gonzales F, Stankunas M, Lindert J, et al. Social support, socio-economic status, health and abuse among older people in seven European countries. PloS one. 2013 Jan; 3. doi: 10.1371/journal.pone.0054856 23382989 PMC3559777

[14] Cochrane. Scoping reviews: what they are and how you can do them. [Online]. [cited 2022 March 3. https://training.cochrane.org/resource/scoping-reviews-what-they-are-and-how-you-can-do-them.

[15] Joanna Briggs Institute; Scoping Reviews Resources. [Online]. [cited 2022 03 3. https://jbi.global/scoping-review-network/resources.

[16] Peters M, Marnie C, Tricco A, Pollock D, Munn Z, Alexander L, et al. Updated methodological guidance for the conduct of scoping reviews. JBI evidence synthesis. 2020 Oct; 18(10). doi: 10.11124/JBIES-20-00167 33038124

[17] Peters M, Godfrey C, McInerney P, Khalil H, Larsen P, Marnie C, et al. Best practice guidance and reporting items for the development of scoping review protocols. JBI evidence synthesis. 2022 Apr; 20(4). doi: 10.11124/JBIES-21-00242 35102103

[18] JBI. JBI Manual for Evidence Synthesis. In.

[19] WHO. Country survey for health workforce capacity of ICU facilities in EMR. World Health Organization; 2021.

[20] Chen S, Chen Y, Feng Z, Chen X, Wang Z, Zhu J, et al. Barriers of effective health insurance coverage for rural-to-urban migrant workers in China: a systematic review and policy gap analysis. BMC Public Health. 2020 Dec; 20(1). doi: 10.1186/s12889-020-8448-8 32228665 PMC7106835

[21] Ouanes-Besbes L, Ferjani M, Abroug F. Intensive Care in Tunisia. ICU Management and Practice. 2017; 17(2).

[22] Ayebale ET, Roche AM, Kassebaum NJ, Biccard BM. Africa’s critical care capacity before COVID-19. Southern African Journal of Anaesthesia and Analgesia. 2020 May; 26(3).

[23] Elhadi M, Msherghi A, Alkeelani M, Alsuyihili A, Khaled A, Buzreg A, et al. Concerns for low-resource countries, with under-prepared intensive care units, facing the COVID-19 pandemic. Infection, disease & health. 2020 Nov; 25(4). doi: 10.1016/j.idh.2020.05.008 32631682 PMC7274573

[24] Lebanese Order of Physicians. [Online].; 2022 [cited 2022 March 03. https://lopbeirut.org/en/homepage-en/.

[25] Elkhayari M, Dilai O, Ziadi A, Hachimi A, Samkaoui MA. Outcome of patients admitted during off hours in Moroccan intensive care unit. International Journal of General Medicine. 2014; 7. doi: 10.2147/IJGM.S54536 24600244 PMC3942112

[26] Saudi Commission for Health Specialties. [Online].; 2015 [cited 2022 02 19. https://www.scfhs.org.sa/MESPS/TrainingProgs/TrainingProgsStatement/Documents/Critical%20Care%20Medicine%20new.pdf.

[27] College of Physicians and Surgeons Pakistan. [Online]. [cited 2022 03 05. https://www.cpsp.edu.pk/fcps.php.

[28] The Libyan Health System: Study of Medical and Allied Health Eductaion and Training Institutions. Ministry of Health Libya, Health Information and Documentation Centre; 2018.

[29] Arab Board of Medical Specializations. [Online].; 2022 [cited 2022 April 27. https://www.arab-board.org/.

[30] The Univsersity of Jordan. [Online].; 2017 [cited 2022 March 01. http://nursing.ju.edu.jo/Lists/OurPrograms/School_Master.aspx.

[31] World Higher Education Database. [Online].; 2020 [cited 2022 01 05. https://www.whed.net/detail_institution.php?KDo2MF0sMzBRLDNEYApgCg = =.

[32] Nayeri ND, Shariat E, Tayebi Z, Ghorbanzadeh M. Challenges of postgraduate critical care nursing program in Iran. Medical journal of the Islamic Republic of Iran. 2017; 31(10). 10.18869/mjiri.31.10 PMC5609326 28955660

[33] Al‐Nassan S, Alshammari F, Al‐Bostanji S, Modhi Mansour Z, Hawamdeh M. Physical therapy practice in intensive care units in Jordanian hospitals: a national survey. Physiotherapy Research International. 2019 Jan; 24(1). doi: 10.1002/pri.1749 30230143

[34] Ebrahim ZH, Ebrahim A. Factors influencing job satisfaction and turnover intention among coronary care unit nurses in Bahrain. International Journal of Nursing & Clinical Practices. 2017; 4(251).

[35] Abbas A, Ali A, Bahgat SM, Shouman W. Prevalence, associated factors, and consequences of burnout among ICU healthcare workers: An Egyptian experience. The Egyptian Journal of Chest Diseases and Tuberculosis. 2019 Oct; 1(68).

[36] Salimi S, Pakpour V, Rahmani A, Wilson M, Feizollahzadeh H. Compassion satisfaction, burnout, and secondary traumatic stress among critical care nurses in Iran. Journal of Transcultural Nursing. 2020 Jan; 31(1). doi: 10.1177/1043659619838876 30957715

[37] Mrayyan MT. Job stressors and social support behaviors: Comparing intensive care units to wards in Jordan. Contemporary nurse. 2009 Feb; 31(1). doi: 10.5172/conu.673.31.2.163 19379118

[38] Hussein J, Aniza I, Taufik JA. Factors associated with organizational stress among intensive care unit healthcare workers, in Somalia hospital. Malaysian Journal of Public Health Medicine. 2012; 12(1).

[39] Eslami K, Aletayeb F, Aletayeb SM, Kouti L, Hardani AK. Identifying medication errors in neonatal intensive care units: a two-center study. BMC pediatrics. 2019 Dec; 19(1). doi: 10.1186/s12887-019-1748-4 31638939 PMC6805622

[40] Alnutaifi N. Alnutaifi N. Knowledge and Skills of Cardiopulmonary Resuscitation among Critical Care Nurses in Kuwaiti Hospitals. American Journal of Nursing. 2021; 9(2).

[41] Shahin M, Bakr Al-Waqfi B, Al-Abed H. Perception of medication errors among critical care nurses in Jordanian Hospitals: causes and reporting. International Journal of Current Research. 2018; 10(11).

[42] Batiha AM, Bashaireh I, AlBashtawy M, Shennaq S. Exploring the competency of the Jordanian intensive care nurses towards endotracheal tube and oral care practices for mechanically ventilated patients: an observational study. Global Journal of Health Science. 2013 Jan; 5(1). 10.5539/gjhs.v5n1p203 PMC4776978 23283054

[43] Shahin MA. Improving intravenous medication administration and reducing medication errors among critical care nurses at Jordan University Hospital. Journal of Bioscience and Applied Research. 2019 Aug; 5(3).

[44] Aloush SM, Abdelkader FA, Al-Sayaghi K, Tawalbeh LI, Suliman M, Al Bashtawy M, et al. Compliance of nurses and hospitals with ventilator-associated pneumonia prevention guidelines: a middle Eastern survey. Journal of Nursing Care Quality. 2018 Jul; 1(33). 10.1097/NCQ.0000000000000286 28858912

[45] Hassan ZM, Wahsheh MA. Knowledge level of nurses in Jordan on ventilator‐associated pneumonia and preventive measures. Nursing in critical care. 2017 May; 22(3). doi: 10.1111/nicc.12273 28008700

[46] Khalil NS, Youssef W, Shalaby LM, Moustafa Z. Oncology critical Care nurse’s knowledge about insertion, care and complications of venous Port catheters in Egypt. Adv Practice Nurs. 2017; 2(2).

[47] Taha E. Critical care nurses’ knowledge and practice regarding administration of total parenteral nutrition at critical care areas in Egypt. Critical Care. 2014; 4(13).

[48] Ghorbani A, Sadeghi L, Shahrokhi A, Mohammadpour A, Addo M, Khodadadi E. Hand hygiene compliance before and after wearing gloves among intensive care unit nurses in Iran. American journal of infection control. 2016 Nov; 44(1). 10.1016/j.ajic.2016.05.004 27311508

[49] Arrar AA, Mohammed SJ. Effectiveness of an Educational Program on Nurses’ Knowledge and Practices Concerning Nursing Care for Critically–Ill Patients at Critical Care Units in Misan Governorate Hospitals. Medico-Legal Update. 2020 Jul; 20(3).

[50] Ali F, Seloma Y. Effect of Nursing Educational Guidelines about pulse oximetry on Critical Care Nurses’ Knowledge at a Selected University Hospital in Egypt. International Journal of Novel Research in Healthcare and Nursing. 2019; 6(1).

[51] Rhodes A, Ferdinande P, Flaatten H, Guidet B, Metnitz PG, Moreno RP. The variability of critical care bed numbers in Europe. Intensive care medicine. 2012 Oct; 38(10). doi: 10.1007/s00134-012-2627-8 22777516

[52] Arabi Y, Phua J, Koh Y, Du B, Faruq M, Nishimura M, et al. Structure, organization, and delivery of critical care in Asian ICUs. Critical care medicine. 2016 Oct; 44(10): p. e940–8. doi: 10.1097/CCM.0000000000001854 27347762

[53] Bennett S, Grawe E, Jones C, Josephs S, Mechlin M, Hurford W. Role of the anesthesiologist-intensivist outside the ICU: opportunity to add value for the hospital or an unnecessary distraction? Current opinion in anaesthesiology. 2018 Apr; 31(2): p. 165–71. doi: 10.1097/ACO.0000000000000560 29341963

[54] Mathivha L. ICUs worldwide: An overview of critical care medicine in South Africa. Critical care. 2002 Feb; 6(1): p. 1–3. 11940262 10.1186/cc1449 PMC137393

[55] Fukuda T, Sakurai H, Kashiwagi M. Impact of having a certified nurse specialist in critical care nursing as head nurse on ICU patient outcomes. PloS one. 2020 Feb; 15(2). doi: 10.1371/journal.pone.0228458 32023315 PMC7001939

[56] Chuang CH T P L C L K C Y. Burnout in the intensive care unit professionals: a systematic review. Medicine. 2016 Dec; 95(50). doi: 10.1097/MD.0000000000005629 27977605 PMC5268051

[57] Nakweenda M, Anthonie R, van der Heever M. Staff shortages in critical care units: critical care nurses experiences. International Journal of Africa Nursing Sciences. 2022 Mar;: p. 100414.

[58] Lee A, Cheung Y, Joynt G, Leung C, Wong W, Gomersall C. Are high nurse workload/staffing ratios associated with decreased survival in critically ill patients? A cohort study. Annals of intensive care. 2017 Dec; 7(1): p. 1–9. 28466462 10.1186/s13613-017-0269-2 PMC5413463

[59] Boutou A, Betsiou S, Pitsiou G, Bitzani M, Kioumis I. Nursing errors in ICU and their association with burnout, anxiety, insomnia and working environment: a cross-sectional study. European Respiratory Journal. 2021. PMC10266324 37324040

