# Supplementary material for: Health workforce capacity of intensive care units in the Eastern Mediterranean Region
Source: PLoS One. 2023 Jun 16;18(6):e0286980. doi: 10.1371/journal.pone.0286980 (PMC10275434; doi:10.1371/journal.pone.0286980)
Supplement: S1 File — (DOCX) [file pone.0286980.s003.docx]

Scoping review of Health workforce capacity of intensive care units in the Eastern Mediterranean Region

Electronic search strategy for Google Scholar

| **#** | **Searches** | **Results** |
| --- | --- | --- |
|  | allinthetitle: critical care Afghanistan | 27 |
|  | allinthetitle: intensive care Afghanistan | 31 |
|  | allintitle: bahrain intensive care units | 2 |
|  | allintitle: djibouti intensive care | 1 |
|  | allintitle: egypt critical care | 24 |
|  | allintitle: egypt intensive care nurses | 2 |
|  | allintitle: egypt intensive care staff | 1 |
|  | allintitle: critical care egypt physicians OR nurses OR staff OR specialists OR training OR education OR programme OR program OR evaluation | 6 |
|  | allintitle: iran critical care | 33 |
|  | allintitle: iran intensive care unit nurses | 14 |
|  | allintitle: iranian intensive care nurses | 18 |
|  | allintitle: iranian intensive care physicians | 2 |
|  | allintitle: iran critical care program | 2 |
|  | allintitle: iraqi intensive care | 2 |
|  | allintitle: iraq critical care | 6 |
|  | allintitle: jordan intensive care | 34 |
|  | allintitle: critical care Jordanian | 20 |
|  | allintitle: critical care Jordan physicians OR nurses OR staff OR specialists OR training OR education OR programme OR program OR evaluation | 5 |
|  | allintitle: critical care lebanon physicians OR nurses OR staff OR specialists OR training OR education OR programme OR program OR evaluation | 4 |
|  | allintitle: Kuwait intensive care | 16 |
|  | allintitle: libya intensive care | 19 |
|  | allintitle: morocco critical care | 1 |
|  | allintitle: morocco intensive care | 18 |
|  | Oman “intensive care” OR “critical care” “ICU” “staff” “nurse” “doctor” “healthcare worker” OR “health workforce” -resistant | 56 |
|  | Oman "job satisfaction" "environment" "nurse" OR "doctor" OR "healthcare worker" | 2,980 |
|  | Pakistan “intensive care” OR “critical care” “ICU” “staff” “nurse” “doctor” “healthcare worker” OR “health workforce” -resistant | 189 |
|  | Pakistan “intensive care” OR “critical care” “ICU” "training" "doctor" "nurse" "service" | 1,290 |
|  | Pakistan "doctor" "nurses" “health workforce" "review" "education" "training" "capacity building" | 613 |
|  | Qatar “intensive care” OR “critical care” OR “ICU” “staff” “nurse” “doctor” “healthcare worker” OR “health workforce” -resistant | 103 |
|  | Qatar “intensive care” OR “critical care” OR “ICU” “training" "education" "staff" "knowledge" -resistant | 2,440 |
|  | Saudi Arabia “intensive care” OR “critical care” “ICU” “staff” “nurse” “doctor” “healthcare worker” OR “health workforce” -resistant | 180 |
|  | Saudi Arabia “intensive care” OR “critical care” "ICU" “training" "education" "experience" "qualification" “knowledge” -resistant | 485 |
|  | Saudi Arabia “intensive care” OR “critical care” OR "ICU" "staff" "nurse" "tertiary" "knowledge" "experience" "training"-resistant | 3,300 |
|  | Syria “intensive care” OR “critical care” “ICU” “staff” “nurse” “doctor” “healthcare worker” OR “health workforce” -resistant | 1,990 |
|  | Syria "healthcare worker" "training" "education" "knowledge" "staff" | 301 |
|  | Somalia “intensive care” OR “critical care” “ICU” “staff” “nurse” “doctor” “healthcare worker” OR “health workforce” -resistant | 33 |
|  | Somalia "health workforce" "training" "education" "knowledge" "staff" | 1,250 |
|  | Yemen “intensive care” OR “critical care” “ICU” “staff” “nurse” “doctor” “healthcare worker” OR “health workforce” -resistant | 28 |
|  | Yemen “intensive care” OR “critical care” OR "ICU" “training" "education" "experience" "qualification" "knowledge" | 307 |
